# Alice’s Delirium: A Theatre-based Simulation Scenario for Nursing

**DOI:** 10.7759/cureus.2411

**Published:** 2018-04-02

**Authors:** Jennifer Dale-Tam, Glenn D Posner

**Affiliations:** 1 Nursing Education, The Ottawa Hospital; 2 Department of Innovation in Medical Education, University of Ottawa

**Keywords:** delirium, simulation, nursing, falls, nursing education

## Abstract

As an educational methodology, simulation has been used by nursing education at the academic level for numerous years and has started to gain traction in the onboarding education and professional development of practicing nurses. Simulation allows the learner to apply knowledge and skills in a safe environment where mistakes and learning can happen without an impact on patient safety. The development of a simulation scenario to demonstrate the benefits of simulation education methodologies to a large group of nurse educators was requested by nursing education leadership at The Ottawa Hospital (TOH). Since the demonstration of this scenario in the fall of 2016, there has been significant uptake and adaptation of this particular scenario within the nursing education departments of TOH. Originally written to be used with a simulated patient (SP), “Alice” has since been adapted to be used with a hi-fidelity manikin within an inpatient surgery department continuing professional development (CPD) program for practicing nurses, orientation for nurses to a level 2 trauma unit and at the corporate level of nursing orientation using an SP. Therefore, this scenario is applicable to nurses practicing in an area of inpatient surgery at varying levels, from novice to expert. It could easily be adapted for use with medicine nursing education programs. The case presented in this technical report is of the simulation scenario used for the inpatient surgery CPD program. Varying adaptations of the case are included in the appendices.

## Introduction

Delirium can occur in 18%-50% of patients in acute care institutions, which can result in increased morbidity and mortality, especially in the elderly [1]. Although delirium is defined as an acute change in mental status from a patient’s baseline cognition, it is often attributed to underlying disease processes or other common contributing factors, such as narcotics used to treat postoperative pain [2]. Many times, delirium is dismissed by nurses. This is consistent with the personal observations of the primary author (JD-T) of nurses practicing in inpatient surgical areas and through discussions with the nurses on the importance of delirium assessment. Therefore, the nursing continuing professional development (CPD) program decided to employ theatre-based simulation as an educational strategy to reinforce the importance of delirium assessment and appropriate interventions.

Falls cost the Canadian healthcare system an estimated two-billion dollars annually and are the leading cause of injury during hospitalization [3]. Falls in the elderly are especially traumatic due to their frailty. For a patient experiencing delirium, their risk of falls increases during their acute confused state; consequently, it was decided to include falls risk assessment as a secondary objective in this theatre-based simulation scenario.

All theatre-based simulation sessions were conducted at the University of Ottawa Skills and Simulation Centre from January 2017 to the end of March 2017 with practicing The Ottawa Hospital (TOH) inpatient surgery nurses of varying years of experience as one sub-session of a larger four-hour nursing CPD session. A total of 63 nurses participated in 20 simulation sessions with two to six participants per session. Each simulation session was one-hour long, consisting of a five-minute pre-briefing, 10 to 15 minutes of simulation, followed by a debriefing of 30 to 40 minutes using the promoting excellence and reflective learning in simulation (PEARLS) framework [4]. Scenario design and implementation were facilitated using the National League for Nursing (NLN) simulation design template [5]. The overall four-hour education session was evaluated, including the simulation session and learning outcomes. The evaluation form is included in Appendix C.

Learning objectives

During the delirium and falls simulation session nurses will:

1.      perform a cognitive assessment using the Cognitive Assessment Method (CAM) tool;

2.     identify delirium;

3.     perform a fall risk assessment;

4.     apply universal fall risk precautions; and

5.     communicate with the patient and the inter-professional team by introducing themselves and using situation, background, assessment, and recommendation (SBAR), as appropriate.

## Technical report

Case summary

Alice Smith is an 83-year-old widow admitted to hospital with a right fractured hip after falling on ice. She has recently been diagnosed with dementia with short-term memory loss and has moved in with her daughter. Ten years ago, she had a myocardial infarction (MI) but is otherwise healthy. She was alert and oriented to person, place, and time on admission last evening. The scenario starts with the primary nurse and a colleague going to do hourly rounding on Alice and repositioning her. She is being kept nil per os (NPO) awaiting surgery. Alice wakes up in a confused state; the nurse(s) should attempt to reorient Alice, perform a confusion assessment method (CAM), identify new-onset delirium, and call the medical team. The nurse should use the SBAR structure to report to the physician and suggest the use of a delirium physician order set. Alice, in her confused state, attempts to climb out of bed; the nurse(s) should complete a fall risk assessment followed by the implementation of universal fall risk precautions.

Personnel

·         Simulation instructor

·         Simulation technician to manage the manikin

·         Confederate physician - if available; otherwise, played by the simulation instructor

·         Alice confederate SP - if available; otherwise, voice played by simulation technician

·         Delirium content expert - if required

Learners

·         Primary registered nurse (RN)

·         Secondary RN

·         Observers

Learner preparation

Using the NLN simulation design template [5], a checklist for the simulation instructor is used for the pre-briefing addressing confidentiality, the purpose of the scenario for formative learning, the orientation to the manikin, simulation room, and equipment, and the assignment of roles. In addition to this checklist, the fiction contract and the basic assumption of simulation (that learners are capable and trying their best) [6] are discussed with the nurses.

Standard high-level objectives are provided to the nurses that are used across all simulations in the inpatient surgery nursing education program during the pre-briefing:

1.      You are to communicate with the patient, your colleagues, and inter-professional team, as appropriate.

2.      You are to assess the patient, identify any issues, and intervene accordingly.

Primary nurse: You are working on the orthopedic surgery floor looking after Alice Smith, who is an 83-year-old widow, recently moved into her daughter’s home after being diagnosed with mild dementia and short-term memory loss. She had an MI 10 years ago. She is admitted with a right hip fracture after falling on ice outside and is NPO awaiting surgery. She was alert and oriented on admission. You are going in with your nursing colleague to do hourly rounding and reposition the patient.

Secondary nurse: You are going in with the primary nurse to reposition Alice and assist where needed.

Observers: You are to observe the simulation, take notes, and prepare to be active participants during the debriefing later.

Set-up

Refer to Appendix A for a detailed equipment list.

The scenario takes place in a single patient room in a tertiary care hospital on an inpatient orthopedic surgery floor.

**Figure 1:**
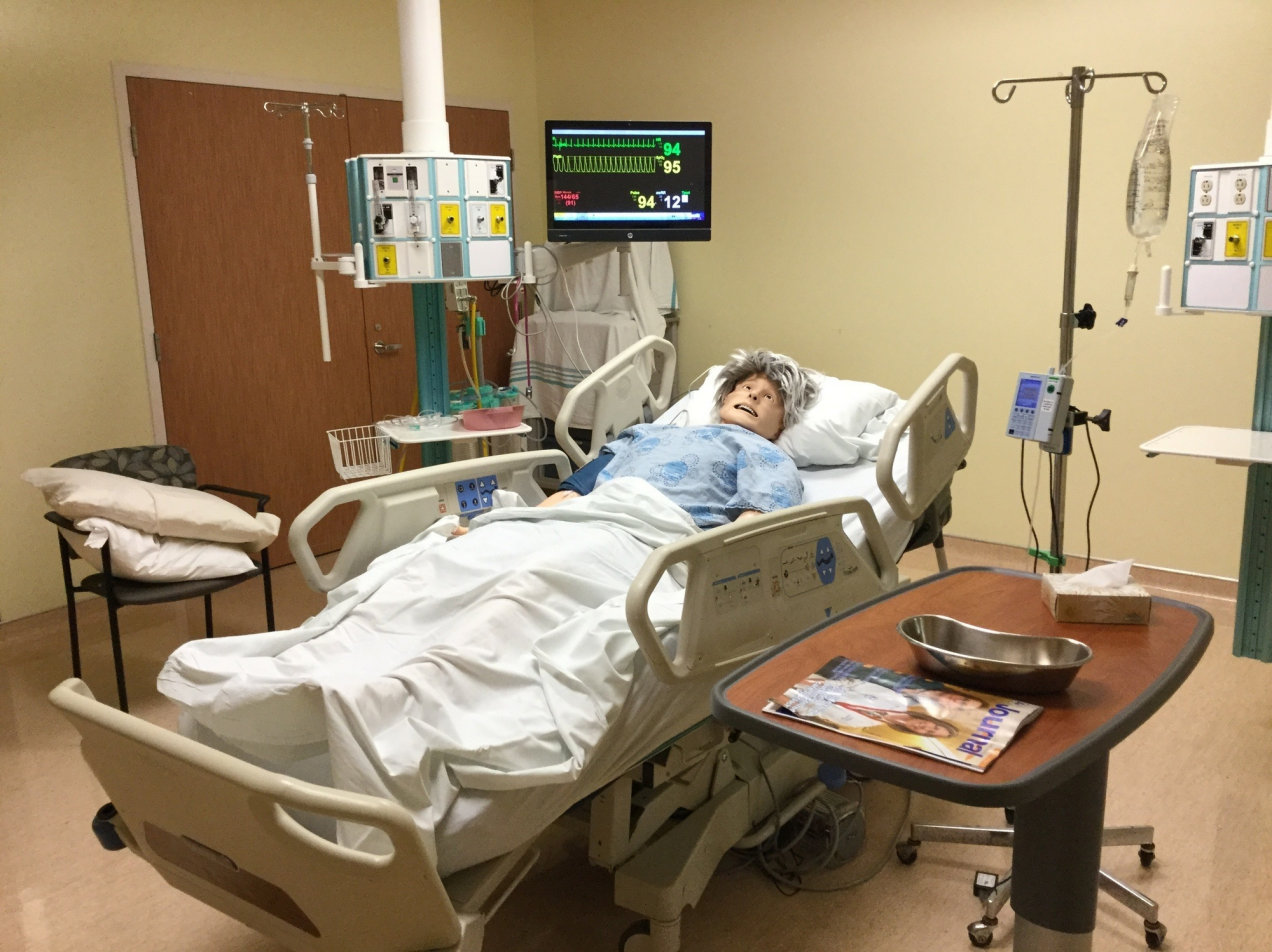
Alice's Room Set-Up Alice set up for a level 2 critical care unit simulation; cardiac monitor, O_2 _saturation monitoring, and blood pressure monitor would not be attached for a standard inpatient room.

A hi-fidelity manikin in a patient gown and an elderly wig is present on an inpatient bed in the simulation room. Intravenous (IV) fluids infusing via an IV infusion pump are attached to the manikin. Standard equipment is available in the room in the form of a suction, an oxygen set-up, and a vital signs monitor (heart rate, blood pressure, temperature, and pulse oximetry). None are attached to the manikin. (Figure 1).

**Figure 2:**
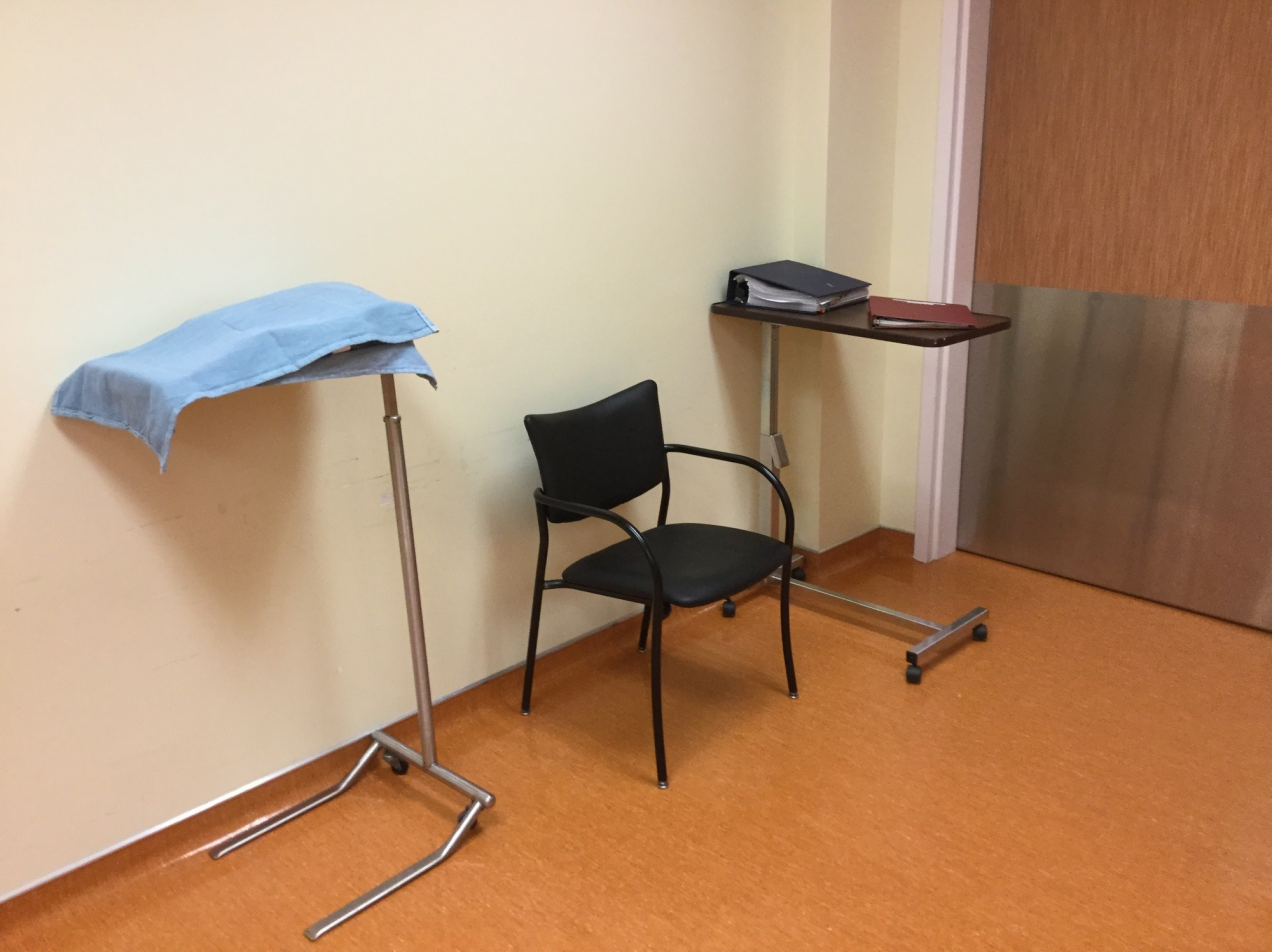
Nursing Station Set-Up Resources covered, made available when requested for during the simulation. Patient chart and policy and parental manual available.

**Figure 3:**
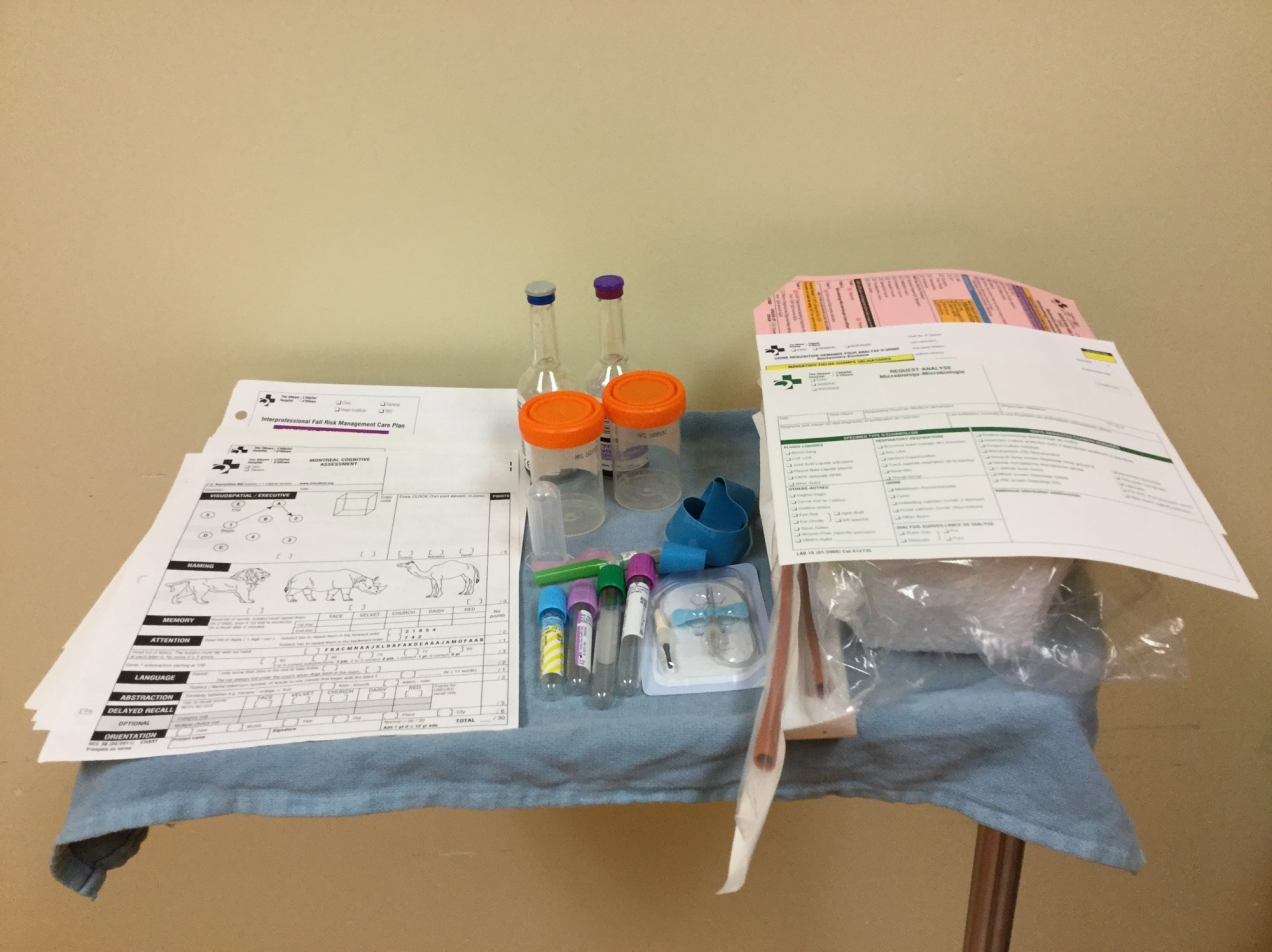
Resources Uncovered Cognitive assessment tools and supplies, as listed on the delirium order set.

A nursing station is set up in a separate area or outside the simulation room. A full patient chart is available at the nursing station. Paper copies of the fall risk assessment and delirium hospital policies are also available at the nursing station in a "policy binder." A fall risk assessment tool and CAM assessment tool become available at the nursing station when a need for use is identified during the simulation, both tools remain hidden; otherwise, the purpose of the simulation would be revealed ahead of time (Figure 2-3).

Scenario progression

The ideal progression is listed in Table 1, including expected interventions by the nurses. Table 2 includes instructor notes along with triggers to advance the scenario when nurses are not progressing toward the learning objectives of the simulation.

**Table 1:** Scenario Progression NPO: Nil Per Os; CAM: Confusion Assessment Method; RNs: Registered Nurses; MD: Medical Doctor; SBAR: Situation, Background, Assessment, Recommendation

Timing (approximate)	Manikin Programming & Actions	Expected Interventions
0 minutes	Alice sleeping	Primary and secondary RNs enter room
1 minutes	Alice sleeping; Heart Rate 95, Blood Pressure 145/65, Oxygen Saturation 94%, Temperature 37.2	RNs to wake Alice, introduce selves, do hourly rounding tasks
2 minutes	Alice wakes up, opens eyes, states “Leave me alone!” “What do you want?” “I need to go make some coffee!”	RNs to reassure and reorient Alice, and explain NPO status
3 minutes	Alice to state “I need to get out of this bed!” “My kids are asleep in a bed like this in the basement; I need to check on them”	RNs suspect delirium
4 minutes	Same mental state Stays in bed when told to by RN	RNs perform CAM assessment
5 minutes	Mumbling “Where’s Spot? I want Spot! Let me out of bed to get Spot”	Same
6-7 minutes	Alice to say: “I really need to get out of this bed. I have to take Spot for a walk. I HAVE to get out of this bed NOW”	RNs identify Alice is in a delirium and pages MD RN to answer phone and give report to MD using SBAR, suggest the use of delirium order set Identify that patient is a fall risk Attempt to settle Alice
7-8 minutes	Alice settles Alice continues to mumble “Where’s my breakfast?”	RNs gather equipment for blood work and urine RNs apply universal fall precautions –puts up stop sign, updates care board Communicates to Alice she is a fall risk
9-10 minutes	Same	Continue care for patient

**Table 2:** Suggested Prompts to Advance Scenario CBC: Complete Blood Count; C&S: Culture and Sensitivity

Time (approximate)	Nurse Actions	Trigger Actions
0-2 minutes	If RN does a set of vital signs	Preprogrammed vital signs appear on the monitor
3 minutes	RN does not suspect or identify delirium	Alice becomes more confused, and agitated. Alice: “Where is George; he is always here in the morning?”
6-7 minutes	RN does not suggest delirium order set	After report from RN, MD (calling on phone from the control room): “sounds like a new delirium; could you please initiate the following orders on the delirium order set –CBC, electrolytes, Urea, Creatinine, Chest X-ray, Urine C&S, and have the pharmacist re-evaluate her meds in the morning?”
7-8 minutes	RN does not identify Alice is a fall risk	Alice states: “Take me out of this jail right now. I don’t need these bars. I’m going to call the police.”

Debriefing

The debriefing should be conducted by an experienced simulation instructor with expertise in the area of delirium management.  If the simulation instructor does not have the knowledge or expertise for a delirium assessment and intervention, along with a fall risk assessment and intervention, a content expert should be present for the simulation and debrief to close any knowledge and performance gaps. It is also advocated that debriefing follow a sound framework. Within the inpatient surgery nursing education program at TOH, the PEARLS framework [4] is used for all debriefing sessions.

## Discussion

Historically, simulation has been used for low-frequency, high-impact patient cases due to the limited availability of such cases in the clinical environment for medical and nursing education. The outcomes of such cases can have a significant impact on patient morbidity and mortality. Simulation can also be used for high-frequency; high-impact cases, as was exemplified with Alice. Nurses who participated in this simulation scenario consistently dismissed the importance of delirium identification and intervention, attributing it to Alice's underlying dementia and use of narcotics to treat her pain. Nurses would focus on the agitated, confused state of Alice, attempting to calm her with reassurance; some would call the doctor for an order of medication, such as haloperidol, to sedate her during the simulation. Sedating medications can potentiate the delirium state, especially in the elderly, masking the underlying cause of the delirium [1]. Performance gaps related to the importance of identification, investigation, and treatment of delirium were addressed during the debriefing. After reviewing the evaluation forms that the nurses completed, a consistent theme of learning emerged: "Delirium is a medical emergency," which was not previously recognized during discussions between clinical nurses on the inpatient units and JD-T. Examples of learning, as stated by the simulation participants, can be found in Table 3.

**Table 3:** Frequency of Learner Comments on the Evaluation Form

Examples Learner comments	Frequency on Evaluation Forms
Delirium is a medical emergency	13
Existence of a delirium order set	6
Not to assume delirium is confusion	1

The fall risk assessment and identification objective was not commonly reached in this simulation; this may be attributed to the use of a hi-fidelity manikin. When Alice is a simulated patient played by a confederate such as in the demonstration for TOH Nurse Educators in 2016 or when used in corporate nursing orientation, the fall risk assessment and identification were more consistently performed. The use of a simulated mobile patient where Alice attempts to climb out of bed triggers the nurse to perform a fall risk assessment. For the simulation using the hi-fidelity manikin, the learning objective and performance gap for the fall risk assessment was discussed and closed in the debrief. A change in patient's cognition makes a patient a fall risk [7]. For adaptations using a simulated confederate patient, see Appendix A.

Nurses self-allocated to either active participant in the simulation or an observer role when groups were larger than four in the inpatient surgery CPD program. Observers were given instructions to watch the simulation, take notes, and prepare to actively participate in the debrief. Observers did participate in the debrief but not as actively as the nurses who were in the simulation. It has been noted that observers who are given a checklist with performance criteria based on the learning objectives experience a similar amount of learning as those who participate in the simulation [8]. An observer checklist was piloted, with a group of four during a simulation session of Alice for a level 2 trauma unit orientation in February 2018.  Evaluation results are in Table 4. See Appendix B for a sample checklist for Alice.

**Table 4:** Evaluation of Observer Checklist

Question	Mean Score (1 - Strongly Disagree to 5 - Strongly Agree)
Were you engaged in the simulation using the checklist?	4.25
The observer checklist was beneficial	4.25
The checklist helped me participate in the debrief	4.75

Evaluations of the inpatient surgery nursing CPD simulation session

A total of 63 nurses attended the sessions with 61 completing evaluations anonymously. Nurses tended to agree that the simulation session was beneficial, with a mean score of 4.72 on a 5-point Likert scale, where 1 was not worthwhile and 5 was beneficial. Many of the nurses have been through simulation sessions both in their nursing training along with previous inpatient surgery CPD days. Prior to the implementation of the Alice scenario, previous simulation sessions subscribed to the low-frequency, high-impact model of application, but the nurses appreciated having a more common, less stressful situation for their simulation session (Table 5). Participation in simulation-based education sessions is stress-inducing to learners [9]. It is hypothesized that using a high-frequency, high-impact goal for simulation scenario design could be used to gain buy-in with participants who have little experience with simulation, as it would be a more familiar scenario seen in the clinical environment.

**Table 5:** Comments on Evaluation Forms

Comments by Active Participants & Observers	
Very realistic, +++learning	Was nice to have a familiar common scenario to reflect on and think about
Good experience	Very realistic situation
Low acuity allowed good focus on basic communication and assessments (CAM)	I like the simulation, was an in-depth overview of a common situation

## Conclusions

The design and implementation of a high-frequency, high-impact delirium and falls risk assessment theatre-based simulation scenario for nursing has been discussed. Adaptations for both manikin-based and simulated patient scenarios have been included. Originally designed as a showpiece case for the use of simulation education methodologies in nursing education to a large group of nurse educators new to simulation, Alice has become entrenched within nursing education in various programs at TOH, which is a large academic healthcare institution.

## Appendices

Appendix A

Equipment List: Manikin-based Simulation

·         IV pump

·         patient chart

·         IV tubing

·         confusion Assessment Method document

·         IV bag of normal saline

·         fall risk assessment document

·         hi-fidelity manikin

·         delirium preprinted order set

·         patient gown

·         related nursing/institutional policies

·         ID bracelet

·         patient bed and linen

·         elderly wig

·         vital signs monitor

·         urinary catheter insertion kit

·         various blood collection tubes

·         urinary catheter

·         urine specimen container

·         blood collection kit

·         bedside table

Equipment List: Simulated Patient

·         Same as above without the hi-fidelity manikin

Additional Equipment Required and Information for a Level 2 Critical Care Monitoring Unit

·         cardiac monitor

·         to be added to the pre-brief, case summary, and patient chart: Alice has a liver laceration requiring close monitoring.

Background Information for the Person Playing “Alice”

History of mild dementia

Confused at present (during the simulation)

Does not need to make sense when answering nurse’s questions posed to her

Trying to get out of bed; has broken right hip

Continually communicates with the RN when asked

Knows her name only

**Table 6:** Alice's Extra Script

General verbal comments:	
“It’s 1961”	“I have to go to school today, I need to find mama”
“It’s June”	“The sky is green”
“Get out of here!”	“I can hear elephants coming!”
“Where’s George?”	“I want my tuba!”

When Alice is played by a confederate, the secondary objective of a fall risk assessment is more effectively achieved when Alice tries to climb out of bed. The safety of the confederate and simulation participants must be kept in mind at all times; Alice should not actually fall.

Appendix B

Instructions: Observe the simulation, complete the checklist, and include any comments. Use this checklist to participate in the debrief after the simulation; the simulation instructor will ask for your feedback.

Learning Objectives for the Simulation

During the delirium and falls simulation session nurse(s) will:

1. Communicate with the patient and the inter-professional team by introducing themselves and using situation, background, assessment, and recommendation (SBAR) as appropriate.

2. Perform a CAM assessment and identify delirium.

3. Perform a fall risk assessment.

4. Apply universal fall risk precautions.

**Table 7:** Observer Checklist

Task		Comments
Introduces self to patient	Yes or No	
Uses SBAR to give report to MD	Yes or No	
Repeats orders back to MD	Yes or No	
Performs a CAM assessment	Yes or No	
Performs a Fall Risk assessment	Yes or No	
Implements universal fall risk precautions: Bed at lowest height Orient Alice to environment and equipment Call bell and personal effects within reach 1-2 side rails up Clear communication Assess for pain	Yes or No	

Appendix C

Civic Campus 4-Hour Teaching Session Evaluation                    Date:         

Using the following 5-point scale:

             Beneficial   5 ----- 4 ----- 3 ----- 2 ----- 1    Not worthwhile

Rate the following sessions:

Presentations:

Accreditation                        5              4             3             2              1

Communication                    5              4             3             2              1

Comments about the above:

_____________________________________________________________

Chest tube skills station:                   5              4             3             2              1

Comments about the above:

_____________________________________________________________

Trach skills station:                             5              4             3             2              1

Comments about the above:

_____________________________________________________________

Traction skills station:                        5              4             3             2              1

Comments about the above:

_____________________________________________________________

Post-mortem skills station:         5         4             3             2              1

Comments about the above:

_____________________________________________________________

Simulation station:                     5          4             3             2              1

Comments about the above:

_____________________________________________________________

Do you feel that the content of the teaching day will provide you with additional knowledge for your clinical practice?             Yes                                No

Please comment why or why not:

________________________________________________________________________________________________

Please list three things you learned from the day.

________________________________________________________________________________________________

Please provide suggestions for material/topics to be reviewed on the unit. ________________________________________________________________________________________________
